# Comparison of oxygen supplementation in very preterm infants: Variations of oxygen saturation features and their application to hypoxemic episode based risk stratification

**DOI:** 10.3389/fped.2023.1016197

**Published:** 2023-02-27

**Authors:** Pravitha Ramanand, Premananda Indic, Colm P. Travers, Namasivayam Ambalavanan

**Affiliations:** ^1^ Department of Electrical Engineering, University of Texas at Tyler, Tyler, TX, United States; ^2^ Department of Pediatrics, University of Alabama at Birmingham, Birmingham, AL, United States

**Keywords:** preterm infants, oxygen supplementation, oxygen saturation signal, sample entropy, generalized multiscale entropy, poincare plot indices, intermittent hypoxemia, risk stratification

## Abstract

**Background:**

Oxygen supplementation is commonly used to maintain oxygen saturation (SpO_2_) levels in preterm infants within target ranges to reduce intermittent hypoxemic (IH) events, which are associated with short- and long-term morbidities. There is not much information available about differences in oxygenation patterns in infants undergoing such supplementations nor their relation to observed IH events. This study aimed to describe oxygenation characteristics during two types of supplementation by studying SpO_2_ signal features and assess their performance in hypoxemia risk screening during NICU monitoring.

**Subjects and methods:**

SpO_2_ data from 25 infants with gestational age <32 weeks and birthweight <2,000 g who underwent a cross over trial of low-flow nasal cannula (NC) and digitally-set servo-controlled oxygen environment (OE) supplementations was considered in this secondary analysis. Features pertaining to signal distribution, variability and complexity were estimated and analyzed for differences between the supplementations. Univariate and regularized multivariate logistic regression was applied to identify relevant features and develop screening models for infants likely to experience a critically high number of IH per day of observation. Their performance was assessed using area under receiver operating curves (AUROC), accuracy, sensitivity, specificity and F1 scores.

**Results:**

While most SpO_2_ measures remained comparable during both supplementations, signal irregularity and complexity were elevated while on OE, pointing to more volatility in oxygen saturation during this supplementation mode. In addition, SpO_2_ variability measures exhibited early prognostic value in discriminating infants at higher risk of critically many IH events. Poincare plot variability at lag 1 had AUROC of 0.82, 0.86, 0.89 compared to 0.63, 0.75, 0.81 for the IH number, a clinical parameter at observation times of 30 min, 1 and 2 h, respectively. Multivariate models with two features exhibited validation AUROC > 0.80, F1 score > 0.60 and specificity >0.85 at observation times ≥ 1 h. Finally, we proposed a framework for risk stratification of infants using a cumulative risk score for continuous monitoring.

**Conclusion:**

Analysis of oxygen saturation signal routinely collected in the NICU, may have extensive applications in inferring subtle changes to cardiorespiratory dynamics under various conditions as well as in informing clinical decisions about infant care.

## 1. Introduction

Intermittent hypoxemic (IH) events, also known as desaturations, are a common occurrence in preterm infants and pose a challenge in maintaining a target oxygen saturation range (1, 2). IH in very preterm infants is associated with multiple short- and long-term morbidities such as retinopathy of prematurity and neurodevelopmental impairment (3). Oxygen supplementation protocols attempt to mitigate such outcomes by stabilizing oxygenation to a desired level and reducing hypoxemic and hyperoxemic episodes (4–6). However, hyperoxemia, resulting from high levels of supplemental oxygen or prolonged exposure to it complicates the situation by potentially leading to oxidative stress and injury (7, 8). The accumulative effect of repeated episodes of hypoxemia/hyperoxemia has been associated with alterations in vascular tone which may injure the vascular bed of organs such as the eyes and the brain (9, 10). Arriving at a suitable range for oxygen saturation in preterm infants which minimizes deleterious health effects is hence challenging and appropriate modes and protocols of oxygen supplementation in this vulnerable population remains an extensively studied topic.

In one such study comparing low-flow nasal cannula (NC) oxygen supplementation (11) with digitally-set servo-controlled oxygen environment (OE) among preterm infants, we demonstrated that use of OE reduced episodes of IH when compared with NC (12). The most significant result of the study was that during OE, both – IH (defined as SpO_2_ < 85% for ≥10 s) and severe hypoxemia (SpO_2_ < 80% for ≥10 s) – were reduced. In addition, the proportion of time in hypoxemia (SpO_2_ < 85%) over 24 h was decreased during OE compared with NC. It was suggested that these improvements in hypoxemic parameters during OE may be due to a more stable hypo-pharyngeal oxygen distribution with this mode of non-invasive oxygen therapy. While hypoxemic parameters showed a difference between OE and NC, the number of bradycardia events did not differ, suggesting that the differences due to treatment modes were to be found in oxygenation patterns rather than in heart rate data.

Studies on oxygen supplementation methods primarily seek to establish appropriate oxygenation levels, check for compliance with target ranges, compare automated vs. manual control of inspired oxygen, enumerate improvements in occurrence of adverse cardio-respiratory events among others (5, 13–15). However, there is insufficient data on the characterization of the oxygenation patterns exhibited by infants undergoing different modes of supplementation. The differences in IH based outcomes between the two supplementations in our study indicated that the dynamic SpO_2_ signal may also exhibit temporal differences during these treatments. Moreover, characterizing the oxygenation behavior may help to better understand the observed reduction of hypoxemic burden during OE compared with NC. In this work, our objective was to describe the dynamic features of oxygen saturation in infants during OE and NC, in greater detail by examining features related to the distribution, variability, irregularity and complexity of these data. Deriving the differences in SpO_2_ features between the two modes of supplementation may explain how these are related to the observed differences in the hypoxemic variables.

Frequency of hypoxemia events has been determined to depend on various factors including gestational age, low birth weight, lower baseline SpO_2_ and exhibits a lot of variability over the first few weeks of post-natal life (2). Oxygen supplementations regularize breathing and reduce instabilities in blood oxygenation levels that result in lower hypoxemic burden in preterm infants, while also preventing occurrence of hyperoxemic episodes (6). In the NICU, caregiving staff are often confronted with multiple alarms across the unit requiring attention, limiting their time for sanitation procedures potentially increasing risk of infection in the infants (16). Predicting hypoxemia or concomitant cardiorespiratory events such as apnea/bradycardia or risk stratification of infants based on their occurrence burden can be helpful in designing interventions to prevent these and provide better care and management in clinical settings (6, 17).

The challenge of predicting adverse events such as apneas which are precursors of most desaturations in preterm infants using cardio-respiratory signals and/or demographic and clinical variables with methods drawn from machine learning and statistical modeling is well presented in Lim et al. (18). As a preliminary step to predicting the occurrence of the events themselves, in this work, we considered the risk stratification of infants who experienced a critically high number of IH per 24 h of observation in the NICU. While this can be accomplished by enumerating the IH events themselves, our intent was to assess the potential of SpO_2_ signal characteristics in early risk screening of infants, prior to the aggregation of such events in each infant. We determined the shortest observation time from the start of monitoring to arrive at such a risk assessment and proposed a framework for continuous risk assessment using developed models that can be easily implemented in the NICU during supplementations.

In this secondary analysis of oxygen saturation data collected during a cross over trial of two supplementation methods, we hypothesized that observed differences in IH events between the supplementations will also be present in the oxygenation patterns of infants. We surmised that variability measures of the SpO_2_ data will differ between the interventions, as hypoxemia are a subset of severe desaturations from baseline levels and were found to be fewer in time and duration during OE. We tested this by estimating a comprehensive set of SpO_2_ measures describing the signal histogram, variability, irregularity, and complexity and deriving differences between them during OE and NC supplementations. In addition, we examined these features for their discriminative ability in screening infants likely to exhibit a critically high number of IH during a 24-h monitoring period and whether such screening performance was enhanced during one type of supplementation over another. The shortest observation time to provide a stratification and a framework for continuous risk screening was also proposed. A pulse oximetry-based model which can risk stratify infants for hypoxemic burden in real time during NICU monitoring may have far reaching applications in informing clinical care and support for this vulnerable population.

## 2. Methods

### 2.1. Subjects, data acquisition and pre-processing

The oxygen saturation data were obtained from preterm infants <30 weeks' gestation admitted to the level 4 Regional Neonatal Intensive Care Unit at the University of Alabama at Birmingham as part of a study registered with www.clinicaltrials.gov (NCT02794662). A detailed description of the study protocol and comprehensive clinical information are available in Travers et al. (12). Infants receiving oxygen therapy *via* nasal cannula with flow rates ≤1.0 liter per kilogram per minute or oxygen environment were eligible for inclusion if they met these criteria: off ventilator and/or continuous positive airway pressure support for more than 48 h prior to study entry, in an incubator for thermoregulation. Infants were excluded if they had any of the following: a major malformation, a neuromuscular condition that affected respiration, a terminal illness, or a decision to withhold or limit support. Informed consent was obtained from parents/legal guardians. This study approved by the Institutional Review Board (IRB-UAB) at the University of Alabama at Birmingham, had oversight provided by both IRB-UAB and an observational and safety monitoring board, appointed by the NHLBI.

This single center randomized cross-over pilot study had a 1 : 1 parallel allocation of infants to the order of testing each of the two interventions (oxygen environment or nasal cannula oxygen) using a stratified permuted block design. The random allocation sequence was generated by the Pediatric Research Office at the University of Alabama at Birmingham and Children's of Alabama. All infants enrolled in the study had routine monitoring with oxygen saturation averaging times of 7 s and uniform target saturation ranges of 91%–95% for the duration of the study. The effective FiO_2_ for infants on NC was calculated using standardized charts based on infant weight, set FiO_2_, and cannula flow (19). It was used to swap from nasal cannula to oxygen environment. The effective FiO_2_ was maintained when swapping from oxygen environment to nasal cannula by using the standardized charts. The set oxygen concentration while on OE was the effective FiO_2_. Following a 24-h period on the first intervention, the infants were crossed-over to a 24-h period using the alternate mode. Then, the infants were crossed-over to a 24-h period using the initial intervention before being crossed-over to another 24-h period on the alternate intervention. The infants had a 15–30-min period between supplementations during which data were not recorded. Infants were swapped between modes of oxygen delivery during this period and it allowed adequate time to ensure oxygen saturations were stable on the new set FiO_2_ before restarting the recording. Infants who no longer required oxygen therapy after the end of at least the second 24-h recording period completed the trial without further crossovers.

A sample size of 25 infants was required to determine if oxygen environment decreased the number of intermittent hypoxemia episodes by 20% in the 48 h crossover period on either intervention, with a power of 80%, a two tailed type I error rate of 0.05, assuming a standard deviation of 0.5 of the mean. Vital signs data were collected using ixTrend (iexcellence, Wildau, Germany) software to electronically capture real time numeric data sampled each second. There were 90 records of data available for analysis, as some subjects did not complete the third and fourth intervention periods.

### 2.2. Data analysis – descriptive measures of SpO_2_ data

The data were preprocessed prior to analysis. Recording errors caused missing values in the SpO_2_ data which were identified from the simultaneously recorded time vector. Together, these missing values formed less than 1% of the total length of the available data for each subject.

**Figure 1:**
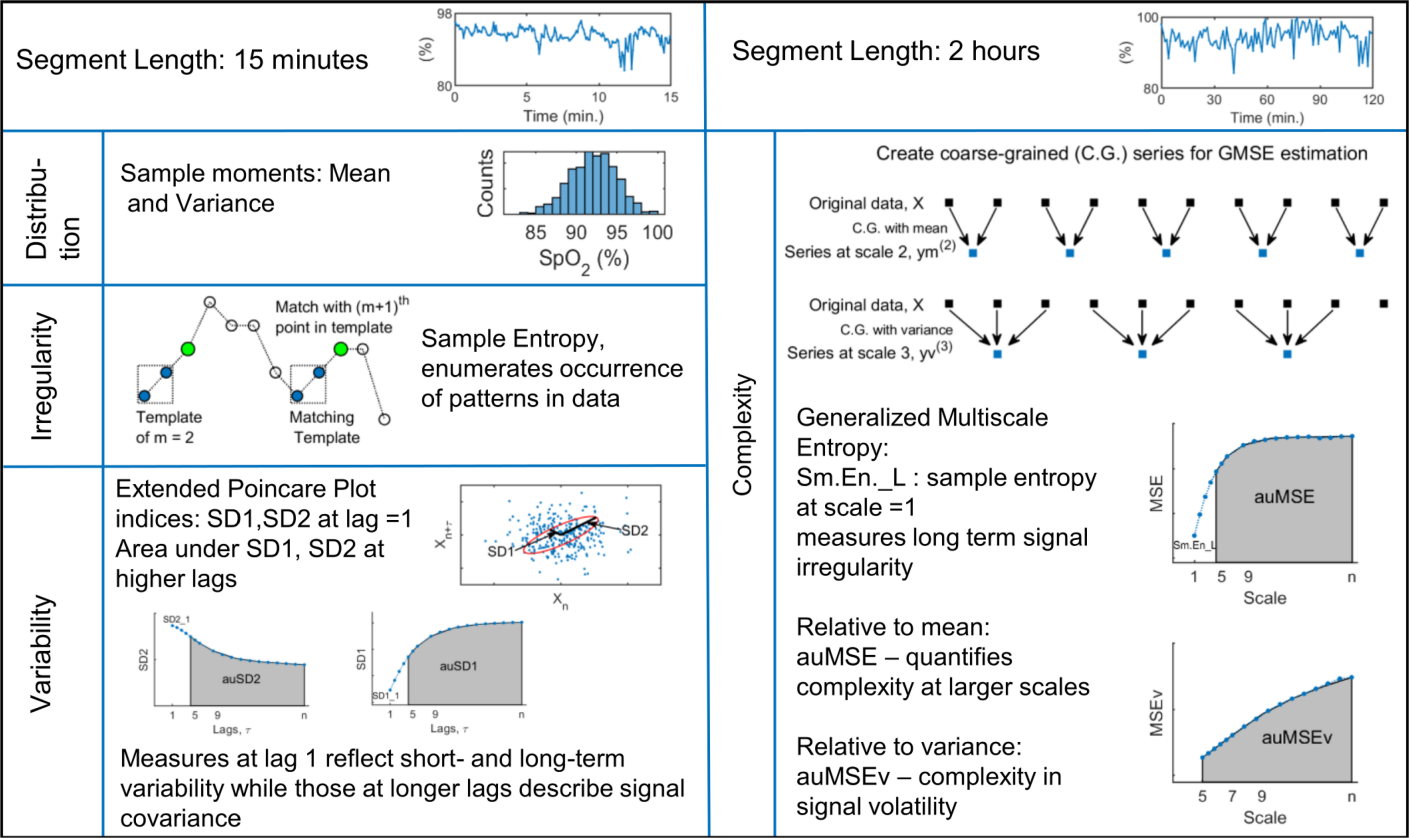
Schematic of the descriptive measures of SpO_2_ data analyzed in this study.

Oxygen saturation data from each day of monitoring were analyzed by estimating descriptive measures over non-overlapping intervals of 15 min and 2 h to capture dynamic behavior over short and long time scales. Data segments with more than 5% missing values were excluded from the analysis. For each subject, the estimated measures were averaged over all available windows to give the estimate over 24 h. In addition, signal measures were averaged over 4 h to give measures at 6 time points spread over the 24 h. Signal behavior related to the histogram, variability, irregularity, and complexity was examined using the sample moments, extended Poincare plot, sample entropy and generalized multiscale entropy measures respectively. These indices have been applied to a variety of physiological and biomedical signals to assess numerous developmental as well as pathology related conditions in infants such as neurodevelopment (20), adverse outcome risk prediction (21, 22), sleep stage classification (23), heart rate variability (24–26), cardiorespiratory dynamics (27), and regulation (28, 29). A brief description of the measures is given next and a schematic of the signal indices is presented in Figure 1.

#### 2.2.1. Signal distribution

Based on common guidelines related to SpO_2_ processing (30), data were described using the mean and variance, the first and second moments of the histogram over 15 min intervals that took into account the inherent non-stationarity of biomedical signals.

#### 2.2.2. Descriptors of variability

The Poincare plot is a scatter diagram in the Cartesian plane with the signal at a given instant plotted against its value from the previous instant (31). The resulting point cloud in phase plane is oriented about axes rotated 45° to the original axes and is characterized by measures describing its distribution in the 2D plane. SD1 refers to the standard deviation about the line of identity and comprises the short-term variability of the signal. SD2 is a measure of the long-term variability of the signal and is the standard deviation perpendicular to the line of identity. An ellipse can be imagined overlying the point cloud with SD1 and SD2 as the semi-minor and semi-major axes respectively. Traditionally, Poincare plot analysis has been applied to heart rate variability data at lag 1, to study the serial correlations within the series (31, 32). This approach has also been extended to include higher lags and applied to different physiological time-series to gain information about temporal correlations and develop screening tools for patients with conditions such as liver cirrhosis and asthma (33).

In this study, we analyzed the Poincare plots of the SpO_2_ signal over windows of 15 min and estimated the variability indices at lags of 1–30. For segments with missing data values, appropriate filtering was applied so that the plotted data points correctly maintained the specified lag between them (34). Besides the variabilities estimated at lag 1, we quantified the correlations at higher lags by the area under the SD1 and SD2 curves between lags 5 and 30 as auSD1, auSD2 respectively.

#### 2.2.3. Irregularity and complexity indices

The irregularity of the signal was quantified by the sample entropy measure. It assigns larger values to sequences of greater randomness or disorder and has been applied widely to biomedical signals because of many attractive properties such as being unaffected by short, noisy series with missing data points and having relative consistency over a range of input parameters (35). It is computed for a series $x=\left\{x_{1},x_{2},\ldots ,x_{N}\right\}\;$ as

(1) $$Sm.En\left(m,r,N\right)=-ln\left(\frac{A^{m}\left(r\right)}{B^{m}\left(r\right)}\right)$$

where $A^{m}$ and $B^{m}$ are average number of template vectors $X_{m}^{\left(i\right)}\;\left(1\leq i\leq N-m\right)$ of length m and m+1, such that the distance between every pair of vectors is within a tolerance r, and calculated as:

(2) $$d\left[X_{m}^{\left(i\right)},X_{m}^{\left(j\right)}\right]=||X_{m}^{\left(i\right)}-X_{m}^{\left(i\right)}|{|}_{\infty },\;1\leq j\leq N-m\;\mathrm{and}\;j\neq i$$

The generalized multiscale entropy (GMSE) method quantifies the complexity of the dynamics of a set of features of the time series related to local sample moments (36). This procedure first rescales the original time series into non-overlapping windows of length τ and creates a new data series by calculating either the mean (first moment) or the variance (second moment) of the data points within that window. These data series can be represented as,

(3) $$y_{j}^{\left(\tau \right)}=\frac{1}{\tau }\sum_{i=\left(\;j-1\right)\tau +1}^{\;j\tau }x_{j},1\leq j\leq \frac{N}{\tau }$$

for coarse graining by the first moment. The subsequent entropy computation of these series is the original multiscale entropy, MSE introduced by Costa et al. (37). Generalized multiscale entropy of volatility fluctuation, MSEv is the sample entropy estimate of these coarse grained series when the data averaging in Equation (3) is replaced by the unbiased variance of data. These two measures differ in the coarse graining procedure and the lowest scale, τ used to create the rescaled series (36).

Template length in Sm.En computation, m was set as 2, and the tolerance as *r* = 0.2 × standard deviation of the data window (38, 39). Sample entropy was estimated over data windows of 15 min (Sm.En_S) to study signal fluctuations over short time scales. The multiscale measures were estimated over segment lengths of 2 h, with scales from 1 to 30 for MSE and 5 to 30 for MSEv as previously recommended (36). The signal complexity over higher scales was quantified by estimating area under the generalized MSE curves between 5 and 30, as auMSE and auMSEv (38). The sample entropy at scale = 1 in the MSE analysis, Sm.En_L is a measure of long term signal irregularity and was included as one of the dynamic measures. Sample entropy and MSE were calculated using codes available at PhysioNet website (https://physionet.org/) (40).

#### 2.2.4. Statistical analysis

The estimated dynamical measures per 24 h were analyzed with mixed linear models using both random effects (intercept) and fixed effects of TREATMENT (OE, NC), DAYS (1, 2, 3, 4 of receiving treatments) and SEQUENCE (ABAB or BABA, where A is OE, B is NC) to account for the cross-over nature of the data (41). Prior to this, the normality of each measure was checked by the Shapiro-Wilk procedure and if found to be non-normal, a log transformation was carried out. The residuals in each case were confirmed to be normal, following linear modeling. For the measures which were significantly different between treatments, their variations over 4-h time intervals were studied by a linear model as above, which also incorporated TIME (6 levels) as a repeated measure and an additional TREATMENT × TIME effect included in the model. The subject effects were specified using SUBJID, the subjects' IDs and SUBJID × TREATMENT in the repeated statement of the mixed linear model. An *F*-test for the difference in measures between supplementations sliced by TIME levels was carried out. All statistical analyses were completed using SAS 9.4 (Cary, NC, USA).

### 2.3. Pulse oximetry based hypoxemia risk screening

Another objective of this study was to assess the ability of the oxygenation signal indices to identify infants likely to experience a critically high number of IH episodes per day of monitoring in the NICU. We evaluated the discriminative performance of signal features estimated over the first 0.5, 1, 2 and 4 h of recording time to detect infants who experienced greater than a critical threshold, *N*_th_ of events over the whole day. Such infants were labeled as “high-risk” or “1” and the rest were marked as “low-risk” or “0”. IH was defined as an event with SpO_2_ < 85% and lasting for longer than 10 s (12). *N*_th_ was chosen as the 75th percentile of all such events aggregated from subjects’ data on all days of monitoring. Since the threshold is determined by number of events, it is itself a predictor of risk. Hence, we also included the number of IH episodes experienced during the observation interval, HyxNum as a feature in the risk screening models.

SpO_2_ features were first assessed individually for discriminative ability between risk groups using logistic models. We also addressed whether the features exhibited preferential performance depending on the supplementation mode the subject was undergoing on a certain day. Leave-one-subject-out strategy (LOSOCV) was used for cross validation, in which each subject was treated as the test subject for the univariate logistic model developed using data from the rest (22). The set of features was further reduced based on significance of coefficients and then multivariate models with two features were developed by applying lasso regularization (42). In this case, validation was done by holding out all records from 20% of subjects and the whole process was repeated 100 times with replacement. Accordingly, in each trial, records from 5 subjects were included in the test group to ensure there were always 17 to 18 records in the test set. This step was necessary to maintain class balance in the test set as there were some subjects who did not complete all 4 days of the experiment. The data records of subjects remaining after forming the validation set made up the training set.

In the final step, we demonstrated an approach that can be easily adopted in a real time screening scenario for infants exhibiting high levels of IH. A composite risk score was derived, that incorporated the screening scores of a subject over previous observation points along with the one developed using data from the current time interval. The final screening was carried out using this weighted cumulative probability score. If *s_i_* denotes the probability score obtained from a model developed using data from an observation interval *i*, the composite score, $CS_{n}$ for the *n*^th^ interval is determined as,

(4) $$CS_{n}=\frac{\sum_{i=1}^{n}w_{i}s_{i}}{\sum_{i}^{n}w_{i}}$$

where $w_{i}$ is the weight or fraction applied to scores, *s_i_*.

**Figure 2:**
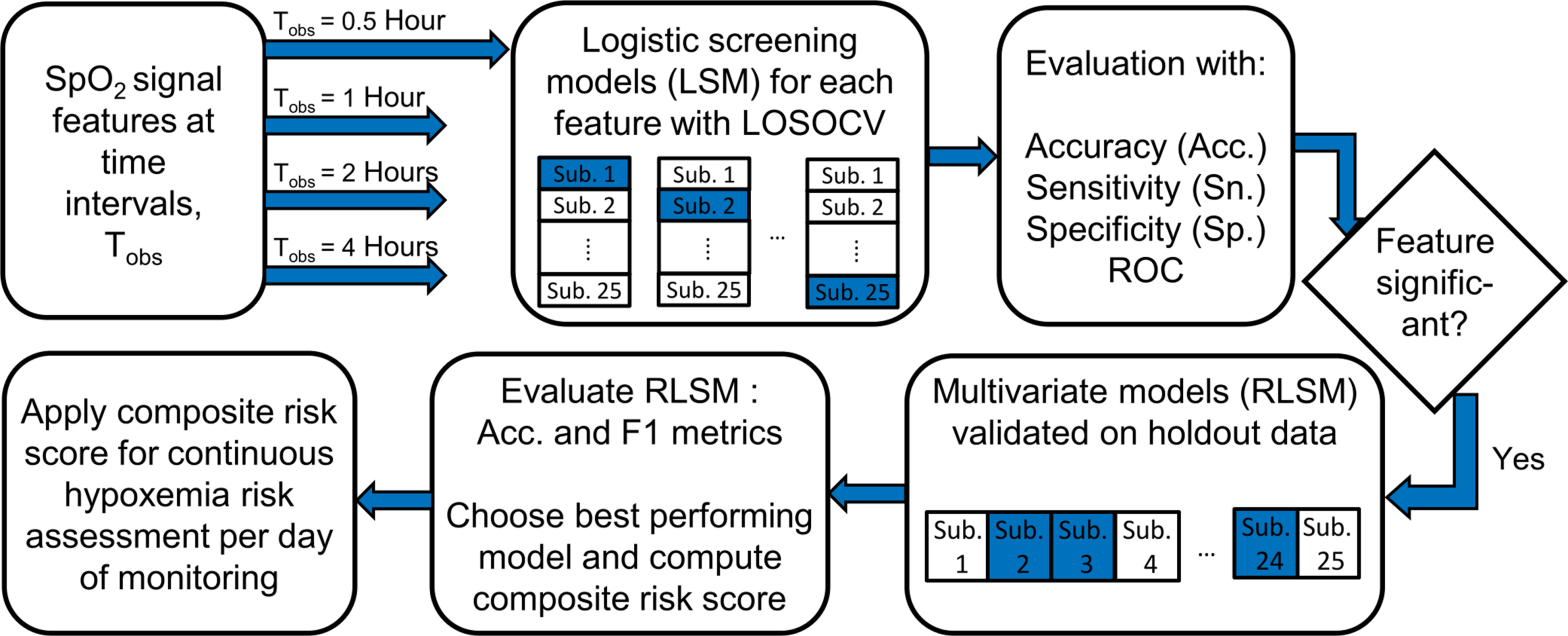
Schematic representation of intermittent hypoxemia (IH) risk screening methodology.

Performance of the univariate- and regularized logistic models was evaluated by comparing results from the developed models and the actual labels using confusion matrices. Several metrics associated with different aspects of performance were calculated as: sensitivity (Sn.) - proportion of correctly classified high-risk infants among all high-risk infants, specificity (Sp.)- proportion of correctly classified low-risk infants to all low-risk infants, accuracy (Acc.) -proportion of all correctly classified infants, positive predictive value (PPV) – proportion of correctly classified high-risk infants among all those classified as high-risk, F1 score- the harmonic mean of precision (PPV) and recall (Sn). In addition, the area under the ROC curve (AUROC), a plot between the sensitivity and (1-specificity) is used as a measure of discriminating ability and a bootstrap with 1,000 replications was used to derive 95% confidence intervals for this measure. Figure 2 outlines the IH risk screening procedure described here. A chi-square test for equality of proportions was used to check whether signal features had better classification performance for data from one supplementation method over the other. The preprocessing, signal measures estimation, model development and validation were all carried out using MATLAB R2021b (Natick, Massachusetts, USA).

## 3. Results

### 3.1. Study participants

**Figure 3:**
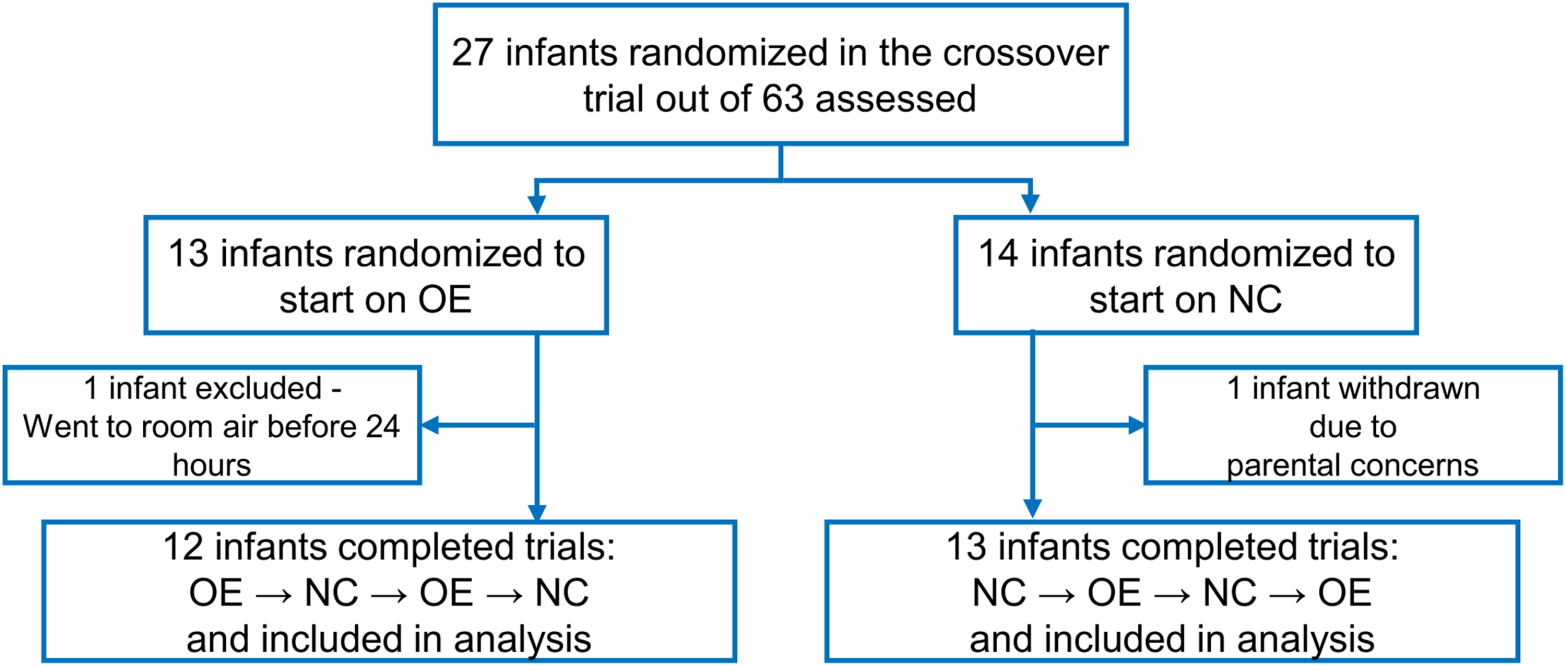
Flow diagram showing screening, randomization, and the number of infants included in the final data analysis. OE is oxygen environment; NC is nasal cannula.

Twenty-seven infants were randomly assigned to the order of intervention. Two infants were excluded from the analysis. One infant was excluded due to withdrawn consent and the other infant was weaned to room air before completing the first 24-h period on oxygen environment. Twenty-five infants completed the study. Figure 3 displays a flow diagram of the randomization and inclusions to data analysis from the study cohort. Eighteen infants completed 96 h of the study, 4 infants completed 72 h, and 3 infants completed 48 h of the study as they were no longer on supplemental oxygen at 48 h.

A set of demographic and clinical characteristics of the infants is given in Table 1. The main outcomes from the primary analysis of this experiment are also included. While both the IH number and proportion of time spent in hypoxemia were reduced while on OE, the proportion of hyperoxic time was comparable between supplementations. In addition, the number of FiO_2_ adjustments were fewer in OE while there was no difference in the effective FiO_2_ level during either intervention.

**Table 1:** Baseline and clinical characteristics of study participants (N =25) and outcomes by mode of oxygen supplementation.

**Demographic and clinical characteristics**		
Male gender, no. (%)	14(56)	
Race		
White, no. (%)	18(72)	
Black, no. (%)	5(20)	
Hispanic, no. (%)	2(8)	
Gestational age, weeks and days	27 3/7 (23 3/7 – 30 2/7)	
Birth weight, grams	1010 (480 – 2080)	
Days after birth at study entry	25 (4 – 86)
Weight at study entry, grams ± SD	1220 (800 – 2380)	
Days ventilated before study entry, days (range)	10 (0 – 47)	
Days on respiratory support, days (range)	64 (6 – 153)	
**Outcomes of primary study per 24 hours**	**OE**	**NC**
*Number of episodes of Intermittent Hypoxemia	98 (4 – 335)	136 (16 – 252)
*Proportion of time spent in Hypoxemia, SpO_2_ < 85%	0.04 (0 – 0.14)	0.06 (0.01 – 0.13)
Time spent in Hyperoxemia, SpO_2_ > 95%	0.18 (0.02 – 0.55)	0.21(0.04 – 0.61)
*No. of FiO_2_ adjustments	5 (0 – 11)	7 (0 – 11)
Effective FiO_2_ during study	0.24(0.21 – 0.38)	0.25(0.21 – 0.43)

Values given as median (min -max) and number (%) where applicable. *Denotes significant differences (p<0.05) between modes of supplementations.

### 3.2. Analysis of dynamic oxygen saturation measures

The oxygen saturation data was characterized by data histogram descriptors, Poincare plot variability indices, sample entropy and multiscale entropy measures. Detailed description of these estimations and their behavior in this cohort is provided in the Supplementary Information Sheet.

**Figure 4:**
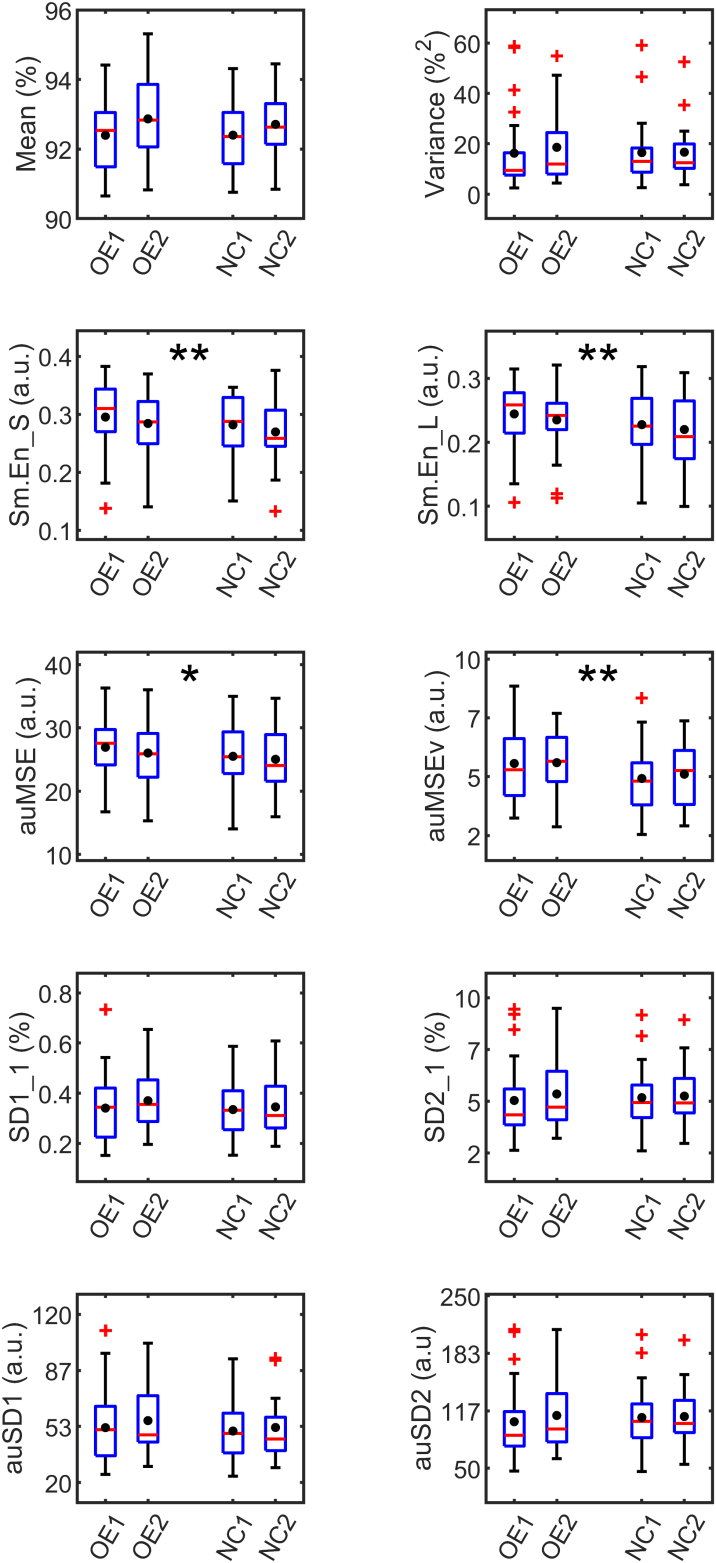
Boxplots of SpO_2_ measures averaged over 24 h grouped by supplementation. OE1,2 and NC1,2 refer to first and second applications of oxygen environment (OE) and nasal cannula (NC) treatments respectively. The red line in each box represents the median, and the black circle, the mean of the measure. The outliers (red markers) are values more than 1.5 times the interquartile range. The box whiskers extend to the most extreme value not considered an outlier. Significant differences from mixed linear analysis observed between treatments OE and NC are marked as *p < 0.05, **p < 0.01.

SpO_2_ signal measures averaged over the observation period of 24 h were analyzed with mixed linear modeling to derive significant differences between supplementation modes. Four measures – variance, SD2_1, auSD1 and auSD2- were found to be non-normal and log-transformed before linear modeling. The boxplots of the measures, shown in Figure 4, represent the distribution characteristics of each measure estimated for the two applications of each mode of oxygen treatment. Mean and median were very close for most measures but among variability measures, the mean was higher than the median, especially for variance, SD2_1 and the auSD2 measures. This indicated positive skewness of these measures, which was also evident from the long whiskers and outliers, extending from the top of the box for these measures.

The fixed effects related to SEQUENCE and DAYS were non-significant for these models suggesting that there were no cross-over effects from one treatment to the next in the experimental procedure. The irregularity measures, Sm.En_S [*F*(1,60.9) = 7.40, *p* = 0.009], Sm.En_L [*F*(1,61.3) = 7.70, *p* = 0.004], and complexity measures, auMSE [*F*(1,61.4) = 4.77, *p* = 0.03], and auMSEv [*F*(1,60.8) = 9.79, *p* = 0.003] were all significantly higher during OE than NC. Sm.En_S was the only short-term measure that differed between the treatment methods. The higher entropy values in OE pointed to more irregularity in the oxygen saturation levels, both in the short- (15 min) and long- (2 h) time scales when compared with NC. The multiscale area measures, comprising the entropy estimates over a range of scales were also found to be higher during OE, when the fluctuations relative to mean and variance were studied. The difference between treatments for the auMSEv measure was greater in magnitude, indicating higher irregularity in the dynamics of local volatility. None of the variability measures differed between supplementations, though auSD1 was just significant with higher value during OE vs. NC (ΔauSD1 = 3, *p* = 0.05).

**Figure 5:**
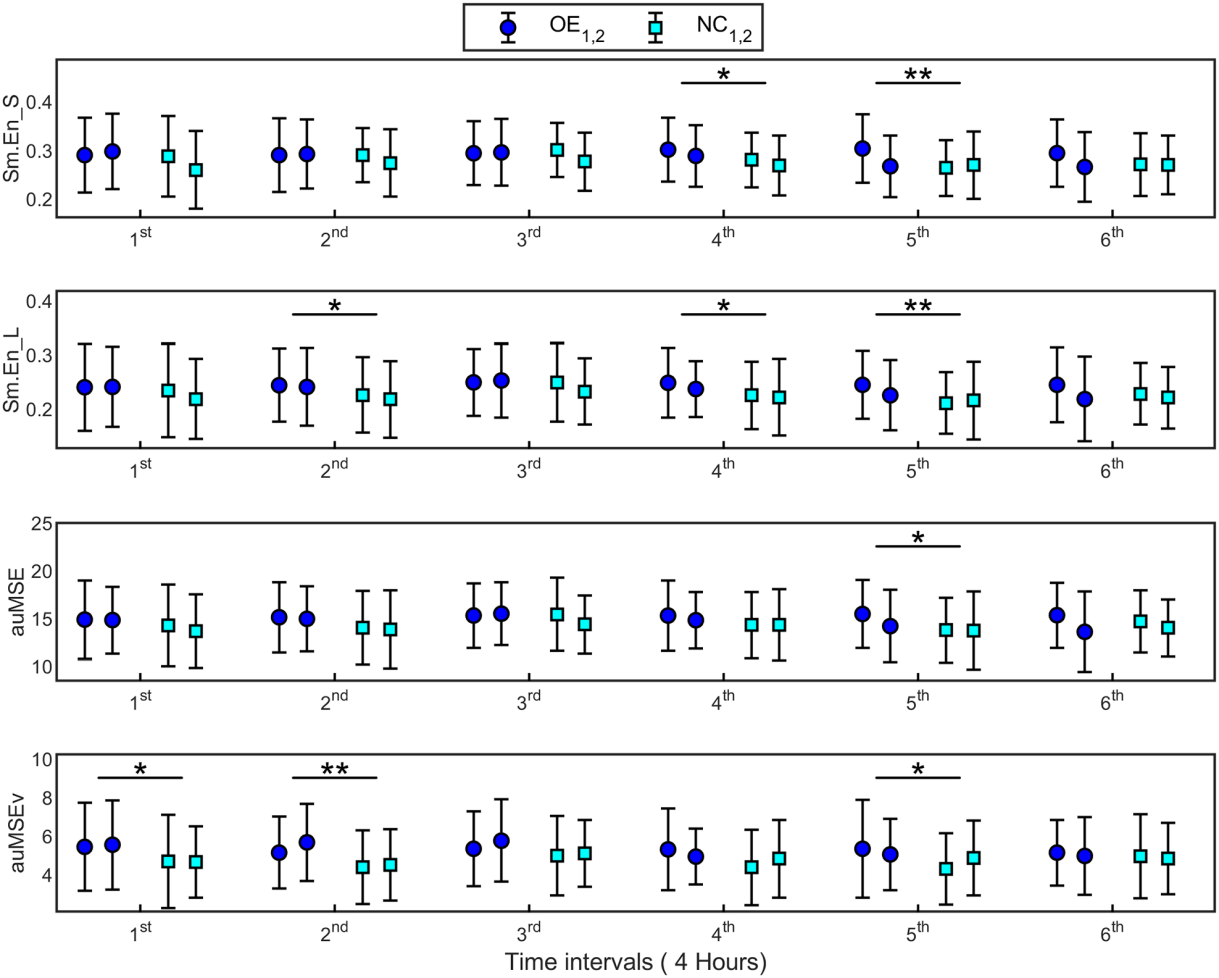
Signal irregularity and complexity measures (mean ± SD) estimated at 6 time points of 4 h each over 24 h, for supplementations OE and NC. Error bars with the same colored marker refer to measures for the first and second applications of the supplementations (OE_1,2_ and NC_1,2_). 1st time interval refers to 0–4 h, 2nd time interval is 4–8 h and so on. The significant differences observed between supplementations at specific time points are marked. *p < 0.05, **p < 0.01.

The significant entropy measures were averaged over non-overlapping intervals of 4 h each and fit with a linear model with repeated TIME (6 intervals) variable, to study their variation over the 24-h monitoring period. In all cases, only the fixed effect of TREATMENT was found to be significant, implying that measures differed between the supplementations. No significant TREATMENT × TIME interaction or repeated TIME effects were found. Hence no differences in the time evolution of measures within each supplementation could be identified. However, the F-test between measures at each time point showed significant differences between supplementations as shown in Figure 5. Up to half the recording time, Sm.En_L and auMSEv were higher during OE compared with NC. Also, while Sm.En_S and Sm.En_L showed differences in the latter part of the 24-h recording, auMSEv was higher during OE even in the first 8 h of supplementation.

### 3.3. Hypoxemia risk screening

Next, we assessed the efficacy of SpO_2_ features from the first few hours of recording (*T*_obs_) in identifying infants having greater than the chosen threshold, *N*_th_, of IH events per day. This threshold was set as 180 events, the 75th percentile of aggregated IH counts. Subject records were labeled “High-risk” or “1” if they had more hypoxemias than *N*_th_ and were assigned “low-risk” or “0” otherwise. It was possible that the same subject was assigned to high and low risk groups on different days of monitoring, based on the number of IH events experienced on a specific day. Hence the risk label was associated with the subject record than with the subjects themselves. The number of subject records with high risk were 22 comprising a little less than quarter (24.4%) of the total records (*N* = 90) analyzed here.

#### 3.3.1. Logistic screening models (LSM)

SpO_2_ measures were assessed using logistic regression models utilizing the LOSOCV methodology and diagnostic accuracy metrics were compiled for each (Table 2). Measures estimated over observation points, *T*_obs_ = 0.5, 1, 2 and 4 h from the start of recording were used to develop LSM. The complexity measures, Sm.En_L, auMSE and auMSEv were estimated over 2-h windows and hence their discriminating ability over shorter observation times were not considered. Comprehensive results from this analysis including those of the chi-square test for the equality of proportion of correctly classified records during each supplementation are available in Supplementary Table S1.

**Table 2:** Performance metrics of logistic screening models (LSM) for hypoxemia risk using SpO_2_ features computed over specific observation intervals, T_obs_. Features with significant models at all T_obs_ are marked in bold.

SpO_2_ Features	Obs. Time in Hours	LOSOCV Metrics				SpO_2_ Features	Obs. Time in Hours	LOSOCV Metrics			
		Acc.	Sn.	Sp.	AUROC (95% CI)			Acc.	Sn.	Sp.	AUROC (95% CI)
Mean	0.5	0.73	0.00	0.97	0.44 (0.28,0.58)	**Poincare plot SD1 at lag=1, SD1_1**	0.5	0.84	0.59	0.93	0.82 (0.67,0.91)
	1	0.73	0.05	0.96	0.52 (0.39,0.67)		1	0.84	0.59	0.93	0.86 (0.73,0.94)
	2	0.73	0.00	0.97	0.49 (0.35,0.63)		2	0.86	0.64	0.93	0.88 (0.77,0.95)
	4	0.73	0.09	0.94	0.55 (0.38,0.68)		4	0.87	0.73	0.91	0.90 (0.78,0.96)
**Variance**	0.5	0.84	0.50	0.96	0.77 (0.60,0.90)	**Poincare plot SD2 at lag=1, SD2_1**	0.5	0.86	0.55	0.96	0.78 (0.61,0.89)
	1	0.86	0.50	0.97	0.80 (0.63,0.91)		1	0.84	0.55	0.94	0.83 (0.67,0.92)
	2	0.80	0.32	0.96	0.81 (0.66,0.90)		2	0.81	0.45	0.93	0.84 (0.73,0.92)
	4	0.84	0.50	0.96	0.89 (0.77,0.95)		4	0.88	0.64	0.96	0.90 (0.80,0.96)
**Short-term Sample Entropy, Sm.En_S**	0.5	0.73	0.23	0.90	0.66 (0.51,0.78)	**Area under Poincare plot SD1 curve, auSD1**	0.5	0.83	0.59	0.91	0.83 (0.68,0.92)
	1	0.73	0.36	0.85	0.70 (0.56,0.81)		1	0.89	0.68	0.96	0.86 (0.72,0.95)
	2	0.73	0.36	0.85	0.67 (0.52,0.79)		2	0.84	0.59	0.93	0.86 (0.74,0.94)
	4	0.76	0.36	0.88	0.70 (0.56,0.81)		4	0.89	0.73	0.94	0.89(0.74,0.97)
Long-term Sample Entropy, Sm.En_L	2	0.70	0.05	0.91	0.57 (0.43,0.70)	**Area under Poincare plot SD2 curve, auSD2**	0.5	0.86	0.55	0.96	0.77 (0.60,0.89)
	4	0.69	0.05	0.90	0.60 (0.45,0.73)		1	0.84	0.55	0.94	0.82 (0.68,0.92)
Area under multiscale entropy, auMSE	2	0.71	0.05	0.93	0.58 (0.45,0.70)		2	0.81	0.45	0.93	0.83 (0.71,0.91)
	4	0.69	0.09	0.88	0.60 (0.44,0.72)		4	0.84	0.50	0.96	0.89 (0.79,0.95)
Area under variance based multiscale entropy, auMSEv	2	0.76	0.00	1.00	0.03 (0.01,0.08)	Number of IH in T_obs,_ HyxNum	0.5	0.72	0.18	0.90	0.63 (0.47,0.77)
							1	0.73	0.27	0.88	0.75 (0.60,0.85)
	4	0.76	0.00	1.00	0.06 (0.03,0.13)		2	0.76	0.45	0.85	0.81 (0.72,0.89)
							4	0.83	0.59	0.91	0.89 (0.80,0.95)

Acc. –Accuracy, Sn. – Sensitivity, Sp. – Specificity, AUROC – Area under ROC

Variability measures - variance, SD1_1, SD2_1 and areas under the SD1 and SD2 curves- exhibited high discrimination between risk groups, with AUROC [mean (SD)] 0.82(0.05), 0.87 (0.03), 0.84(0.05), 0.86(0.02), 0.83(0.04) over the 4 observation intervals accordingly. These features had validation AUROC > 0.75 even at *T*_obs_ = 0.5 h with the lower bound of the confidence interval over 0.5, the value for a random classifier. For *T*_obs _< 2 h, the clinical HyxNum parameter had lower AUROC than the Poincare plot measures. While specificity from the variability features based models was quite high (∼0.95) at the commonly used classification threshold of 0.5, the sensitivity was found to be quite low with median (min, max) of 0.38(0.18, 0.47). When a lower threshold was set at 0.4, we obtained higher sensitivity 0.55(0.32, 0.68) for these models, with minimal decrease in specificity (Table 2 and Supplementary Table S1). In comparison, HyxNum had sensitivity of 0.18 at *T*_obs _= 0.5 h and a maximum of 0.59 at *T*_obs_ = 4 h at the lower classification threshold. At *T*_obs_ = 4 h, SD1_1, SD2_1 and auSD1 exhibited sensitivities of 0.73, 0.64, 0.73 respectively (Table 2).

The test of equal proportions of accurate classifications had insignificant differences for most signal features at observation intervals less than 2 h. This meant that signal features performed equivalently on validation data from the two supplementations (Supplementary Table S1). At *T*_obs_ = 2 h, auSD1 was the sole predictor with significantly higher proportion during OE. However, this behavior was not consistently exhibited. For the 4 h observation interval, auSD1 had equal accuracy in classifying segments from OE and NC, whereas SD1_1 and HyxNum showed increased accuracy during OE. Most signal features did not show preferred predictive ability during a treatment mode, which led us to disregard the mode of supplementation in further modeling analysis.

#### 3.3.2. Regularized logistic screening models (RLSM)

In the next step, multiple logistic regression models with two predictors were developed to improve the screening performance of univariate models by including more predictors, while simultaneously ensuring that data were not overfit, by regularizing using LASSO. At each *T*_obs_, only features whose LSM provided significant AUROC values (lower bound of the 95% CI > 0.5) were included to develop RLSM. Mean, Sm.En_L, auMSE, auMSEv measures were hence excluded as potential predictors. RLSM were built on training data consisting of records from 80% of subjects while the remaining were reserved as test cases. Proper stratification of high-/low- risk cases in the two data sets was ensured (23.5% in test set and 24.7% in training) and the validation process was repeated 100 times for each *T*_obs_. In all trials, although RLSM were significantly better than a constant model, they were considered further only if at least one of the predictors reached significance. More information regarding RLSM such as the features selected, their occurrence statistics and performance metrics are provided in Supplementary Table S2.

The number of significant RLSM models increased with observation time. At *T*_obs_ < 2 h, the most frequently occurring models had short-term sample entropy and Poincare plot SD1 as features with F1 scores reaching a maximum of 0.85 and AUROC of over 95%. At *T*_obs_ = 2 and 4 h, SD1_1 paired with HyxNum to provide comparable performance. As *T*_obs_ increased from 30 min, trials with variance as one of the model features, altered to include either Sm.En_S or HyxNum as the variable that paired with SD1_1 to give significant models. The specificity and accuracy had median values over 85% in all these cases. Models with two variability based features selected together formed the majority of trials, but failed the constraint of significance set for consideration. The rest were significant models having one or three predictors, which were also excluded for reasons of having been already included under LSM (one predictor models) or to avoid overfitting accordingly.

Finally, to demonstrate the use of such screening models in a real time scenario, we considered the RLSM at each *T*_obs_ with the highest F1 score and the highest AUROC < 1 (to avoid overfitting), to classify subjects as high-/low-risk on each day of monitoring. In addition, the final classification was based on the cumulative probability score as in equation (4), by weighing risk scores at each previous time point by 0.6 and the current one by 0.4 to arrive at the composite score. We found that this approach gave incremental accuracies from 87% to 91% and F1 values from 70% to 82% as *T*_obs_ increased from 0.5 to 4 h respectively (Table 3). These F1 scores meant that the sensitivity and PPV of the screening at the shortest half hour window was at least 70% or higher. Performance metrics observed by applying risk scores computed using different combinations of weights are in Supplementary Table S3. For most of these cases, as expected, the classification accuracy and F1 scores improved steadily over longer observation periods.

**Table 3:** Performance metrics of high-IH incidence detection with the cumulative risk score computed using chosen high performing RLSM models at each observation interval, T_obs_

T_obs_ (Hours)	RLSM features	Validation metrics for chosen model					Classification metrics based on cumulative risk score	
		Acc.	Sn.	Sp.	F1	AUROC	Acc.	F1
0.5	Sm.En_S, SD1_1	0.94	0.75	1	0.86	0.96	0.87	0.7
1	Sm.En_S, SD1_1	0.94	1	0.92	0.89	1	0.88	0.73
2	auSD1, HyxNum	0.94	0.75	1	0.86	0.88	0.89	0.75
4	SD1_1, HyxNum	0.94	0.75	1	0.86	0.96	0.91	0.82

Sm.En_S - Short-term Sample Entropy, SD1_1 – Poincare plot SD1 at lag=1, auSD1 – area under Poincare plot SD1 curve, HyxNum- number of IH in T_obs_, Acc. –Accuracy, Sn. – Sensitivity, Sp. – Specificity, F1 – F1 score, AUROC – Area under ROC.

## 4. Discussion

This secondary analysis of oxygen saturation data collected from extremely preterm infants exposed to supplemental oxygen revealed subtle temporal variations in signal measures between two experimental conditions. In addition, we demonstrated the utility of signal features in screening for infants at high risk of having a critically large number of intermittent hypoxemic events per 24 h, as early as the first half hour of monitoring. The predictive value of signal features was not reliant on the supplementation mode, indicating their applicability to hypoxemia monitoring scenarios irrespective of the treatment being administered. Oxygen saturation patterns contain important information regarding differential cardio-respiratory dynamics under various modes of oxygen supplementation and can be utilized for prediction and modeling of intermittent hypoxemia in preterm infants.

Comparing SpO_2_ indices describing different aspects of signal behavior, we found that the signal irregularity and complexity measures were consistently higher during OE supplementation. Besides the sample entropy measures at short- and long-time scales (*p* < 0.01 for both Sm.En_S and Sm.En_L), the multiscale entropy related to the signal mean (*p* = 0.03) and variance (*p* < 0.01) computed over higher scales (from 5 to 30) were also determined to be elevated during OE treatment mode when averaged over the whole recording time. It was noted from time varying analysis of measures, that the generalized entropy related to local volatility (MSEv) remained higher in OE than NC for more than half the recorded time per supplementation. These findings support our hypothesis that oxygenation patterns of infants show distinctive differences when there is varying hypoxemic burden during supplementations. These differences were detected in the irregularity and complexity parameters of the oxygen saturation levels and not the variability parameters as was expected. Signal variability measures, especially those derived from the extended Poincare plot exhibited discriminative ability in identifying infants likely to experience many IH events over the course of a day of monitoring in the NICU. This screening had accuracy >80% within the first half hour of the monitoring.

Our previous study on oxygen supplementation protocols in infants established fewer IH and lesser time spent in them during OE supplementation mode (12). In the present work, we probed how these macroscopic changes were reflected in oxygen saturation levels at various time scales. Descriptors of SpO_2_ data histogram and Poincare plot variabilities remained comparable pointing to dynamical similarities during these treatment modes. Marked differences occurred only relative to signal entropy or randomness, indicating more irregular oxygen saturation during OE. The sample entropy statistic, based on information theory, measures irregularity of signals by quantifying the repeatability of a template in the data series and is especially suited for comparing short, noisy data usually recorded in clinical and biomedical experiments. A low sample entropy indicates more regularity in data with persistent patterns while a higher estimate suggests more randomness (43).

Physiological signals exhibit complex temporal fluctuations not only in the signal itself, but in its local moments which can be captured by the multiscale approach which encompasses scales in addition to the shortest one. We adopted the generalized multiscale approach and studied the signal complexity related to moments of data at longer time scales. This method has been applied to compare normal, healthy conditions as opposed to disease states in adults by analyzing different physiological time series in various fields of medicine and pathology (44). This study is a novel application of the generalized multiscale approach to seek understated changes in oxygen saturation signal variations in infants. A key finding was that the oxygen saturation levels during OE had higher complexity than during NC, relative to both sample moments, and higher irregularity in signal volatility was observed for at least two consecutive 4 h periods over the 24 h monitoring period. Greater signal randomness and volatility may thus be persistent features of oxygenation during OE, though this finding is currently based on the analysis of simple differences and needs to be further validated. Currently, the main focus of studies on oxygen treatment protocols are optimization of time spent in the target oxygenation range and efficacy of treatment on respiratory distress outcomes (45, 46). Findings such as ours may lead to better understanding of signal patterns exhibited during treatments currently administered in infants with respiratory distress, providing clues to their relative merits and efficacies.

The physiological underpinnings of these findings may be on how oxygen saturation is affected by respiratory insufficiency in preterm infants undergoing supplementation and on the immaturity of their cardiorespiratory network (2, 47). Hypoxic events typically follow apneas and are likely enhanced by depletion of pulmonary oxygen stores at low lung volume, decreased blood oxygen carrying capacity, and increased peripheral oxygen consumption (1). A recent study in adults established a stronger causal relationship between SpO_2_ fluctuations and respiratory control during normobaric hypoxia compared to hypoxia, with increasing SpO_2_ sample entropy as the fraction of inspired oxygen, FiO_2_ was reduced (48). The higher irregularity in oxygen saturation levels observed in the present study may be a consequence of the response of an immature cardiorespiratory network to the changes in FiO_2_ during OE supplementation. This can be a direction of future investigation where the behavior of SpO_2_ entropy measures immediately following FiO_2_ adjustments is considered.

In spontaneously breathing infants supported with nasal continuous positive airway pressure, hyperoxemias following FiO_2_ changes (usually increases) were found to be longer in duration than the IH event duration (49). These overshoot hyperoxemias shortened the length of desaturations thereby reducing the overall hypoxemic duration. However, in our case, the proportion of hyperoxemic time was comparable between supplementations. Also, there were fewer FiO_2_ adjustments while on OE, and there was no difference in the effective FiO_2_ concentration between interventions (12). Hence, we cannot currently associate the observed reduction in hypoxemic time while on OE to this effect. On the other hand, the greater irregularity and volatility in oxygen saturation may be acting to prevent sustained desaturations, leading to the reduction in number of events and time spent in IH during OE supplementation. Higher volatility of oxygenation signifies rapidly changing SpO_2_ levels in OE leading to fewer repetitions and/or absence of cyclic changes or patterns. This may prevent the system from remaining in hypoxemic or desaturation states, thus reducing the incidence and duration of clinically relevant IH. An infant's breathing in an oxygen environment, although it more closely mimics spontaneous breathing than with the nasal cannula, may be associated with increased work of breathing due to reduced positive end-expiratory pressure, causing instabilities to the breathing rate, leading to greater fluctuations in oxygen saturation. Another possibility is that an infant's breathing with the nasal cannula apparatus may experience some element of nasal obstruction, which may mildly increase carbon dioxide levels, thus increasing respiratory drive and causing lower oxygen saturation signal entropy and complexity. However, carbon dioxide levels were not recorded in this study. While the specific mechanism needs to be investigated further, this finding suggests that various modes of oxygen administration may modulate the respiratory effort and thereby the underlying cardio-respiratory dynamics during supplementation in preterm infants.

Occurrence prediction of adverse cardiorespiratory events, especially bradycardia and apnea, in infants by varied methodologies such as point process modeling (50), Gaussian mixture models (17) and machine learning (18) is capturing a lot of research interest. Advanced machine and deep learning techniques for hypoxemia prediction among others are also being actively developed (51, 52). It is well appreciated that preterm infants exhibit a lot of variability in their hypoxemic burden in terms of the number of events experienced and the total time spent in them (2, 53). A thorough understanding of the relationship between SpO_2_ signal features and hypoxemic outcomes in infants is a necessary first step before resorting to computationally intensive modeling paradigms. With model brevity in mind, in this study, we derived an SpO_2_ measures-based classifier to screen infants with respect to the number of IH they may experience on any given day of NICU monitoring. We focused on three aspects in this analysis: (1) identifying descriptive features capable of screening infants at risk of having IH greater than a critical threshold, (2) testing whether the predictive performance of these features favored one mode of supplementation over the other, and (3) determining the shortest observation period at the start of monitoring to provide reliable predictions.

Univariate analysis showed that variability measures from extended Poincare plot analysis, SD1_1 and auSD1, distinguished subjects at high risk, as early as the first half hour of monitoring. Both these measures, which quantify correlations in SpO_2_ difference series, outperformed the clinical parameter HyxNum, the IH count for each subject in an observation interval (*T*_obs_). For *T*_obs_ < 4 h, the HyxNum had lower discriminative ability characterized by lower AUROC values when compared with SD1_1 and auSD1. These and other variability measures had higher diagnostic sensitivity and specificity as well, compared with HyxNum, even at observation intervals of 4 h. The variability indices were also chosen in a majority of trials in the RLSM analyses and provided high F1 and specificity among other metrics on validation data. Hence the ability of signal variability measures to identify significant hypoxemia risk even with short observation times is superior to that of conventionally enumerated measures such as the number of observed hypoxemias in the same observation period (HyxNum). This finding suggests that signatures of future desaturations that develop into hypoxemias are detectable in the signal dynamics prior to the aggregation of the events themselves.

The comparison between proportions of correct classifications during OE and NC did not exhibit any preference for either, leading us to cautiously conclude that such screening models may be applied for risk stratification irrespective of the treatments being followed. A framework for real-time screening employing a composite risk score demonstrated progressively increasing accuracy and F1 scores in identifying high-risk subjects as the observation period widened. A risk screening tool as proposed here can be easily incorporated into monitoring devices in the NICU and early warnings of IH risk may be used to initiate interventions to prevent or mitigate their occurrence over subsequent hours of observation. However this methodology is currently only a proof of concept and needs to be validated by adopting interventions in real time based on risk stratification predictions and analyzing the cardio respiratory data following it, as has been done previously in interventional studies to improve breathing instability (54). In this study, we did not investigate the underlying causes or nature of IH events, which are known to be numerous and varied ranging from immature respiratory control (2) to more acute reasons such as abdominal contractions in ventilated preterm infants (55). SpO_2_ variability indices, which show promise in IH risk detection can be assessed for their predictive capability on hypoxemic outcomes in specific scenarios or arising from a subset of causes.

Our subject sample was limited in size but met the statistical power requirements for the primary objectives described in (12). In addition, it had the advantage of having subjects assessed in a randomized cross-over experimental design which allowed each to act as their own control. The finding of elevated complexity in oxygen saturation patterns during OE poses several interesting research questions. How does greater signal irregularity or complexity contribute to the observed reduction in hypoxemic outcome measures observed in OE, while no differences in signal variability are apparent? Yet another is whether adjustments made to FiO_2_ during the experiment are followed by hyperoxemic overshoots that lead to systematic changes in the signal behavior and if so, are these changes consistent across treatment protocols. More research along these lines, will help elucidate the response mechanisms involved in stabilizing infant oxygenation levels by supplementation. In developing the risk screening protocol, we pooled subject data regardless of the different supplementation methods they received, under the supposition that irrespective of treatment differences, a risk stratification of infants for extreme hypoxemic outcomes will inform care in the NICU. This was based on our finding of no difference in the proportions of correct classifications from either mode. While we determined SpO_2_ variability measures as having prognostic value in predicting hypoxemic events, these results will have to be validated on a larger subject sample. IH risk detection using pulse oximetric measures may also find general application in infants undergoing cardio-respiratory monitoring, albeit with rigorous validation in diverse infant groups and scenarios. Tackling these issues and the prediction of the timing of hypoxemia occurrence will be the future directions of this work.

## 5. Conclusions

Analysis of the dynamic measures of oxygen saturation patterns revealed differences in oxygenation patterns between OE and NC oxygen supplementations. SpO_2_ signals had higher irregularity and randomness in volatility while on OE. Greater fluctuations in the blood oxygen levels occurring during OE may be contributing to shorter desaturations, and in turn to fewer clinically relevant hypoxemic events. Contrary to expectation, SpO_2_ variability measures did not exhibit differences between the supplementation modes considered here. On the other hand, the variability measures outperformed all other features in identifying subjects with high levels of intermittent hypoxemia, irrespective of the type of supplementation they were receiving. A model based on these features validated on a bigger cohort will be simple yet effective in early risk stratification of infants susceptible to adverse hypoxemic outcomes. Such models can potentially be useful in clinical settings to modulate care to susceptible infants and avert undesirable cardiorespiratory events.

## Data Availability

The original contributions presented in the study are included in the article/**Supplementary Material**, further inquiries can be directed to the corresponding author.
