# Supplementary material for: Comparison of oxygen supplementation in very preterm infants: Variations of oxygen saturation features and their application to hypoxemic episode based risk stratification
Source: Front Pediatr. 2023 Feb 27;11:1016197. doi: 10.3389/fped.2023.1016197 (PMC10009221; doi:10.3389/fped.2023.1016197)
Supplement: Supplementary file 1 [file Datasheet1.pdf]

Supplementary Material

Dynamical measures of SpO_2 during OE and NC

Measures characterizing the oxygen saturation patterns were estimated over windows spanning 2 time-scales – a short one of 15 minutes and a long one corresponding to 2 hours – for each application of each treatment. The statistical mean, variance, sample entropy and (extended) Poincare plot indices were estimated over shorter windows of 15 minutes to capture rapid changes to these measures whereas multiscale entropy computed at differences scales, was estimated over 2-hour windows. **Figure S1** shows the curves of MSE for each scale and extended Poincare plot measures over lags, averaged over all subjects during OE and NC separately. As observation scale increased, the entropy measures rose sharply for multiscale estimations related to both mean (MSE) and variance (MSEv), in both modes. However, for MSE, it was a slower increase after scale =10, while entropy increased progressively for MSEv (F**igure S1 (A), (B)**). This points to a richer structure related to signal volatility in SpO_2 dynamics under supplementation, irrespective of the treatment mode. The steep rise in MSE over short time scales can be attributed to more rapid fluctuations in the signal mean over this region, after which they are smoothed out by the coarse-graining procedure. On the other hand, the entropy of signal variance remained high at higher time scales signifying that the signal volatility was irregular at longer scales. Between the two treatments, the curves for these measures also diverged beyond scale=10.

The Poincare SD1 curve was visually similar to MSE, increasing up to lag 10 and then exhibiting a slower rise over lags from 11 to 30. The SD2 at increasing lags on the other hand, showed a smoother decrease from its maximum at lag 1 (**Figure S1 (C) and (D)**). Among all these, the OE and NC curves were seen to be well separated for the generalized MSE related to the variance, and for the Poincare SD1 and the MSE functions to a lesser extent. These curves were characterized by the area between scales (and lags) 5 to 30 for the multiscale entropy (and extended Poincare plot) measures. Linear modeling was carried out on these to determine whether the perceived differences were significant statistically and these results are described in section 3.2 of the main text.

Figure S1. Multiscale entropy functions related to the mean and variance at each scale from 1 (5) to 30 ((A) and (B)) respectively. The SD1 and SD2 measures from the Poincare plot at lag values from 1 to 30 are in (C) and (D). The markers are the mean over all subjects during a certain supplementation and the shaded regions represent standard error at each scale/lag. Sm.En- sample entropy, Std. Dev-standard deviation.

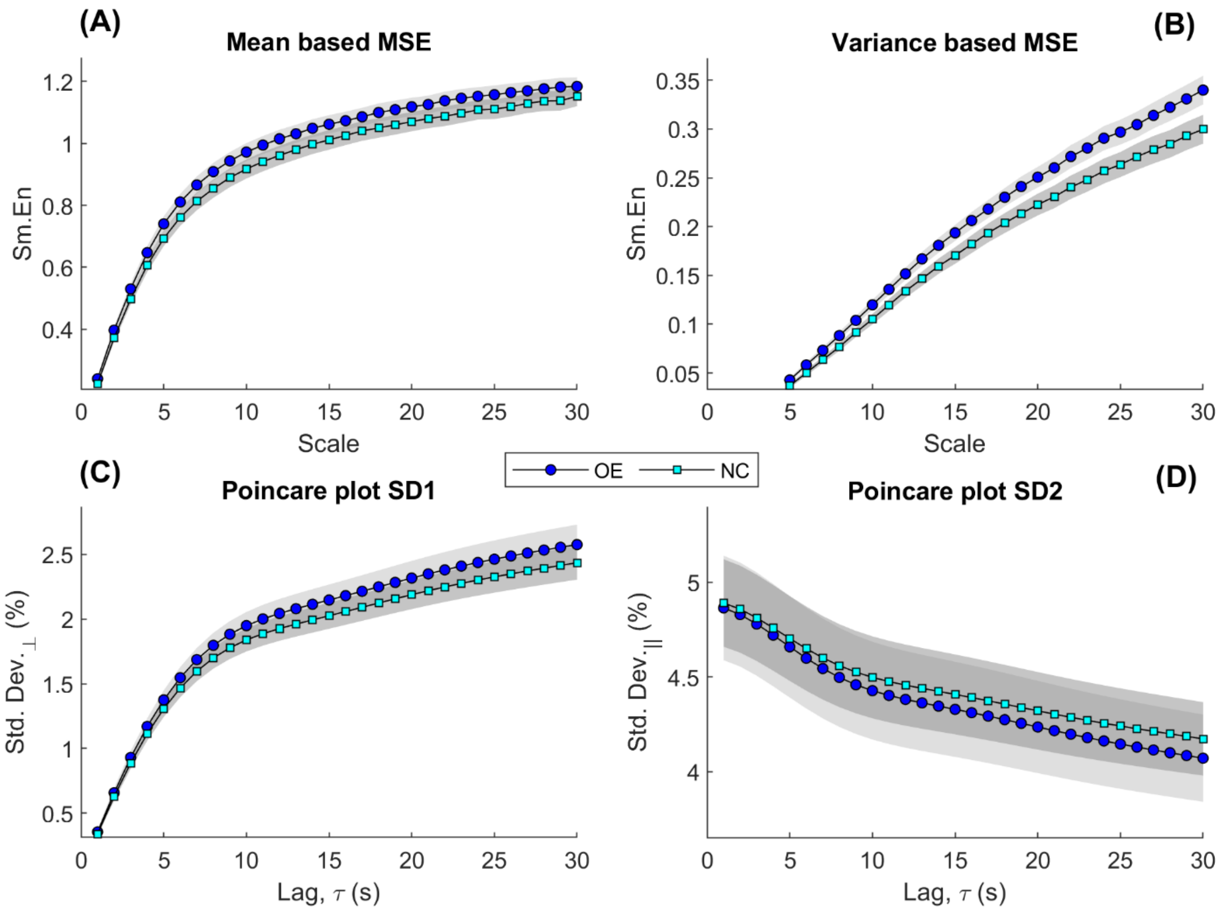

Supplementary table 1 (Table S1): Diagnostic metrics on validation data for LSM developed for each SpO_2 feature estimated over specific observation intervals, T_{obs}. Model coefficients are representative estimates for the feature at the trial when the test data corresponds to the 25th subject. Results of Chi-square test of equality of the proportions of correct classifications between oxygen environment (OE) and nasal cannula (NC) supplementations are also shown.

LSM	Obs. Time (Hou-rs)	Est. Coeff. (SE) for 25th trial	LOSOCV metrics							Comparison between supplementations		
Features			Threshold = 0.5			Threshold = 0.4			AUROC (95% CI)	Prop. Corr. OE	Prop. Corr. NC	p-value
			Acc.	Sn.	Sp.	Acc.	Sn.	Sp.				
Mean	0.5	-0.18 (0.11)	0.76	0.00	1.00	0.73	0.00	0.97	0.44 (0.28,0.58)	0.74	0.77	0.80
	1	-0.21 (0.11)	0.76	0.00	1.00	0.73	0.05	0.96	0.52 (0.39,0.67)	0.74	0.77	0.80
	2	-0.26 (0.15)	0.76	0.00	1.00	0.73	0.00	0.97	0.49 (0.35,0.63)	0.74	0.77	0.80
	4	-0.35 (0.17) *	0.74	0.00	0.98	0.73	0.09	0.94	0.55 (0.38,0.68)	0.74	0.72	0.80
Variance	0.5	0.09 (0.03) **	0.85	0.38	0.96	0.84	0.50	0.96	0.77 (0.60,0.90)	0.89	0.81	0.28
	1	0.07 (0.02) **	0.84	0.33	0.95	0.86	0.50	0.97	0.80 (0.63,0.91)	0.89	0.77	0.11
	2	0.06 (0.02) **	0.79	0.18	0.93	0.80	0.32	0.96	0.81 (0.66,0.90)	0.83	0.74	0.32
	4	0.10 (0.03) **	0.82	0.24	0.93	0.84	0.50	0.96	0.89 (0.77,0.95)	0.85	0.77	0.31
Sm.En_S	0.5	-8.0 (2.9) **	0.75	0.03	0.98	0.73	0.23	0.90	0.66 (0.51,0.78)	0.77	0.72	0.62
	1	-12.1 (3.5) **	0.75	0.08	0.92	0.73	0.36	0.85	0.70 (0.56,0.81)	0.81	0.67	0.15
	2	-11.7 (3.6) **	0.74	0.03	0.93	0.73	0.36	0.85	0.67 (0.52,0.79)	0.77	0.70	0.46
	4	-13.3 (3.8) **	0.77	0.17	0.91	0.76	0.36	0.88	0.70 (0.56,0.81)	0.81	0.72	0.33
Sm.En_L	2	-8.01 (3.3) *	0.75	0.00	0.99	0.70	0.05	0.91	0.57 (0.43,0.70)	0.74	0.74	1.00
	4	-9.1 (3.6) *	0.73	0.03	0.92	0.69	0.05	0.90	0.60 (0.45,0.73)	0.74	0.70	0.62
auMSE	2	-0.09 (0.04) *	0.74	0.00	0.97	0.71	0.05	0.93	0.58 (0.45,0.70)	0.74	0.72	0.80
	4	-0.11(0.04) **	0.70	0.03	0.89	0.69	0.09	0.88	0.60 (0.44,0.72)	0.72	0.67	0.61
auMSEv	2	-0.02(0.09)	0.76	0.00	1.00	0.76	0.00	1.00	0.03 (0.01,0.08)	0.74	0.77	0.80
	4	0.01 (0.11)	0.76	0.00	1.00	0.76	0.00	1.00	0.06(0.03,0.13)	0.74	0.77	0.80
SD1_1	0.5	10.2 (2.4) ***	0.85	0.47	0.92	0.84	0.59	0.93	0.82 (0.67,0.91)	0.89	0.79	0.18
	1	11.3 (2.6) ***	0.86	0.45	0.93	0.84	0.59	0.93	0.86 (0.73,0.94)	0.91	0.79	0.09
	2	13.9 (3.3) ***	0.83	0.41	0.91	0.86	0.64	0.93	0.88 (0.77,0.95)	0.89	0.74	0.06
	4	20.8 (4.8) ***	0.87	0.58	0.92	0.87	0.73	0.91	0.90(0.78,0.96)	0.94	0.79	0.04
SD2_1	0.5	0.59 (0.16) **	0.85	0.38	0.96	0.86	0.55	0.96	0.78 (0.61,0.89)	0.89	0.81	0.28
	1	0.60 (0.15) **	0.85	0.30	0.95	0.84	0.55	0.94	0.83 (0.67,0.92)	0.87	0.81	0.45
	2	0.62 (0.16) **	0.82	0.27	0.93	0.81	0.45	0.93	0.84 (0.73,0.92)	0.85	0.79	0.45
	4	1.01 (0.25) **	0.84	0.31	0.93	0.88	0.64	0.96	0.90 (0.80,0.96)	0.87	0.79	0.30
auSD1	0.5	0.07 (0.02) **	0.83	0.38	0.93	0.83	0.59	0.91	0.83 (0.68,0.92)	0.87	0.79	0.30
	1	0.07 (0.02) **	0.90	0.43	0.96	0.89	0.68	0.96	0.86 (0.72,0.95)	0.94	0.86	0.23
	2	0.07(0.02) **	0.86	0.43	0.93	0.84	0.59	0.93	0.86 (0.74,0.94)	0.94	0.77	0.02
	4	0.12 (0.03) ***	0.91	0.53	0.96	0.89	0.73	0.94	0.89(0.74,0.97)	0.96	0.86	0.11
auSD2	0.5	0.02 (0.02) **	0.85	0.38	0.96	0.86	0.55	0.96	0.77 (0.60,0.89)	0.89	0.81	0.28
	1	0.02 (0.02) **	0.85	0.30	0.95	0.84	0.55	0.94	0.82 (0.68,0.92)	0.87	0.81	0.45
	2	0.03 (0.01) **	0.80	0.23	0.92	0.81	0.45	0.93	0.83 (0.71,0.91)	0.81	0.79	0.83
	4	0.04 (0.01) ***	0.83	0.28	0.93	0.84	0.50	0.96	0.89(0.79,0.95)	0.85	0.79	0.45
HyxNum	0.5	0.23 (0.08) **	0.74	0.15	0.89	0.72	0.18	0.90	0.63 (0.47,0.77)	0.77	0.70	0.46
	1	0.17 (0.05) ***	0.76	0.13	0.89	0.73	0.27	0.88	0.75 (0.60,0.85)	0.79	0.72	0.46
	2	0.14 (0.03) ***	0.78	0.38	0.84	0.76	0.45	0.85	0.81 (0.72,0.89)	0.81	0.72	0.33
	4	0.12 (0.02) ***	0.84	0.50	0.93	0.83	0.59	0.91	0.89 (0.80,0.95)	0.91	0.74	0.03

*p<0.5, **p<0.01, ***p<0.0001 Est. Coeff. (SE)– LSM parameter coefficient (Standard error) for each measure. Acc. –Accuracy, Sn. – Sensitivity, Sp. – Specificity, AUROC – Area under ROC, Prop. Corr. OE(NC) – Proportion of correctly classified OE (NC) records

Supplementary Table 2 (Table S2): The performance metrics of regularized classifier models on validation data. In each of 100 trials, significant RLSM with two predictors are presented. The data are given as median (min, max).

T_{obs} (Hour)	Chosen features	No. of models	Validation Metrics				
			Acc.	Sn.	Sp.	F1-score	AUROC
0.5	Sm.En_S, SD1_1	9	0.82 (0.76,0.94)	0.5 (0.5, 0.75)	0.92 (0.85,1)	0.57 (0.5,0.86)	0.88 (0.67,0.96)
	Variance, SD1_1	3	0.94 (0.94,0.94)	0.75 (0.75,0.75)	1(1,1)	0.86 (0.86,0.86)	1(1,1)
	auSD1, HyxNum	1	0.82	0.5	0.92	0.57	0.79
No. of significant models		13					
1	Sm.En_S, SD1_1	12	0.82 (0.65,0.94)	0.63 (0.5,1)	0.92 (0.69,0.92)	0.62 (0.4,0.89)	0.83 (0.67,1)
	auSD1, HyxNum	10	0.82 (0.76,0.88)	0.25 (0.25,0.25)	1(0.92,1)	0.4(0.33,0.67)	0.78 (0.69,0.88)
	Variance, SD1_1	1	0.70	0.5	0.77	0.44	0.81
No. of significant models		23					
2	SD1_1, HyxNum	20	0.85 (0.71,0.88)	0.5 (0.25,1)	0.85(0.77,1)	0.66(0.29,0.8)	0.91(0.73,1)
	auSD1, HyxNum	14	0.88 (0.82,0.94)	0.5(0.5,0.75)	1(0.92,1)	0.67 (0.57,0.86)	0.85 (0.73,0.90)
No. of significant models		34					
4	SD1_1, HyxNum	31	0.88 (0.71,0.94)	0.75(0.25,1)	0.85(0.69,1)	0.8(0.29,0.86)	0.94(0.85,1)
	auSD1, HyxNum	10	0.88 (0.82,0.94)	0.5(0.5,0.75)	1(0.92,1)	0.67(0.57,0.85)	0.98(0.92,1)
	SD1_1, auSD1	2	(0.76,0.88)	(0.75,0.75)	(0.77,0.92)	(0.6,0.75)	(0.87,0.88)
No. of significant models		43					

T_{obs} – Observation time interval, Acc. – Accuracy, Sn. - Sensitivity, Sp. – Specificity, F1 - F1 score, AUROC – Area under ROC

Supplementary Table 3 (Table S3): Accuracy and F1 scores derived from the composite risk score evaluated by varying the weights applied to scores from models developed for different observation time intervals.

W_1	W_2	T_{obs} = 0.5 hour		T_{obs} = 1 hour		T_{obs} = 2 hours		T_{obs} = 4 hours	
		Acc.	F1	Acc	F1	Acc	F1	Acc	F1
0	1	0.87	0.7	0.88	0.74	0.83	0.63	0.87	0.73
0.2	0.8	0.87	0.7	0.89	0.76	0.86	0.68	0.9	0.79
0.4	0.6	0.87	0.7	0.89	0.73	0.91	0.75	0.9	0.82
0.5	0.5	0.87	0.7	0.88	0.74	0.88	0.73	0.9	0.79
0.55	0.45	0.87	0.7	0.87	0.71	0.88	0.73	0.91	0.82
0.6	0.4	0.87	0.7	0.88	0.73	0.89	0.75	0.91	0.82
0.65	0.35	0.87	0.7	0.88	0.73	0.89	0.75	0.91	0.82
0.7	0.3	0.87	0.7	0.88	0.73	0.89	0.75	0.91	0.82

W_1 – Weight applied to scores from previous intervals, W_2 – Weight applied to score from the current interval, Acc – Accuracy, F1 – F1 score
